# Cancer of the Body of the Pancreas

**Published:** 1909-10

**Authors:** E. S. McKee

**Affiliations:** Cincinnati, O.


CANCER OF THE BODY OF THE PANCREAS.

CHAUFFARD, at a meeting of the Academy of Medicine of Paris, reported three cases of this relatively rare affection. The symptomatology was explained by the anatomical connections which seemed sufficiently precise to make the clinical diagnosis possible. Pain of a special character was the chief symptoms. In two, it started on the left side on a level with costal margin. This pain little by little, extended towards the middle line and was localised in the epigastrium, low down and above the umbilicus, often radiating towards the back, the chest, the shoulders, and took on a very typical character. The paroxysms became more frequent, of longer duration, and very acute, producing a corset-like constriction. The patients adopted a characteristic attitude, only finding a little relief sitting bent slightly forward with the knees bent up, thus relaxing the abdominal muscles as much as possible. No food could be retained during these crises, and between the crises no special dietary seemed to have any preventative action. Intestinal fulness, a false need of going to stool, was a symptom in two cases. In the three cases no tumor could be felt, but in some the existence of a deeply situated hard tumor can be felt in the middle line. There was no enlargement of the supra clavicular or inguinal lymphatic glands and no ascites. Vomiting was rare and jaundice appeared very late. The gall-bladder could not be felt on palpation and the liver was but slightly enlarged. The symptoms of cancer of the body of the pancreas are entirely different from those of the head. In two cases operated upon by Dr. Tuffler, the patient was given enormous relief and thought himself well.—E. S. McKee, M. D., Cincinnati, O.

